# Intact *Mycobacterium leprae* Isolated from Placenta of a Pregnant Woman, China

**DOI:** 10.3201/eid2508.190114

**Published:** 2019-08

**Authors:** Zhiming Chen, Yanfei Kuang, Haiqin Jiang, Wenyue Zhang, Ying Shi, Santosh Chokkakula, Huan Chen, Junhua Li, Hongsheng Wang

**Affiliations:** Chinese Academy of Medical Sciences Institute of Dermatology, Nanjing, China (Z. Chen, H. Jiang, W. Zhang, Y. Shi, S. Chokkakula, H. Wang);; Hunan Provincial Center for Disease Control and Prevention, Changsha, China (Y. Kuang, H. Chen, J. Li);; Jiangsu Key Laboratory of Molecular Biology for Skin Diseases and STIs, Nanjing (H. Wang);; Nanjing Medical University Center for Global Health, Nanjing (H. Wang)

**Keywords:** pregnant woman, placental barrier, Mycobacterium leprae, tuberculosis and other mycobacteria, China

## Abstract

Whether *Mycobacterium leprae* transmits from placenta to fetus remains unknown. We describe the case of a pregnant woman with untreated histoid leproma. Although her newborn was healthy, laboratory examination revealed intact *M. leprae* present in the placenta, suggesting that the placental barrier might prevent vertical dissemination of *M. leprae*.

Leprosy is an infectious disease caused by *Mycobacterium leprae* in susceptible persons. The disease affects the skin and peripheral nerves and, in later stages, can cause irreversible disability. Dissemination of *M. leprae* is thought to occur through nasal mucosa (*1*). However, in pregnant patients, whether *M. leprae* can transmit to the fetus remains unknown. We report the case of a pregnant woman who had histoid leproma and refused therapy until after birth. The Ethics Committee of the Chinese Academy of Medical Sciences’ Institute of Dermatology approved this study, and all persons provided informed consent before sample collection.

In December 2017, a pregnant woman sought care at the Chinese Academy of Medical Sciences’ Institute of Dermatology (Nanjing, China) with a 9-month history of asymptomatic multiple erythema and nodose lesions on her trunk. She had experienced dry skin and dysesthesia in both lower extremities for >10 years. In 2009, she had a sudden rash of erythema on her trunk and lower extremities, which was treated as eczema, without improvement. She began losing her eyebrows in 2015. Her pregnancy was discovered 3 months before admission. Since her illness onset, she had experienced no fevers or joint pain, and her family history was negative for leprosy. 

Physical examination revealed multiple brown papules and firm nodules on her trunk and face (Figure, panels A, B). Superficial sensation was slightly impaired over the lower extremities. No peripheral nerve or superficial lymph node enlargement was observed. Her eyebrows were lost completely. A skin biopsy from her face revealed a subepidermal clear zone, numerous foamy histiocytes throughout the dermis, dense cellularity, and few perivascular lymphocytes. Prominent acid-fast bacilli were observed inside the dermis (Figure, panels C–E). PCR was performed to detect *M. leprae* DNA fragments of *RLEP* and *FolP1*. Samples from a facial lesion tested positive. Serologic examination of the patient’s peripheral blood using ELISA was positive for antibodies of NDO-BSA (IgM), MMP-II (IgG), and LID-1 (IgG) (Appendix Table 1).

**Figure:**
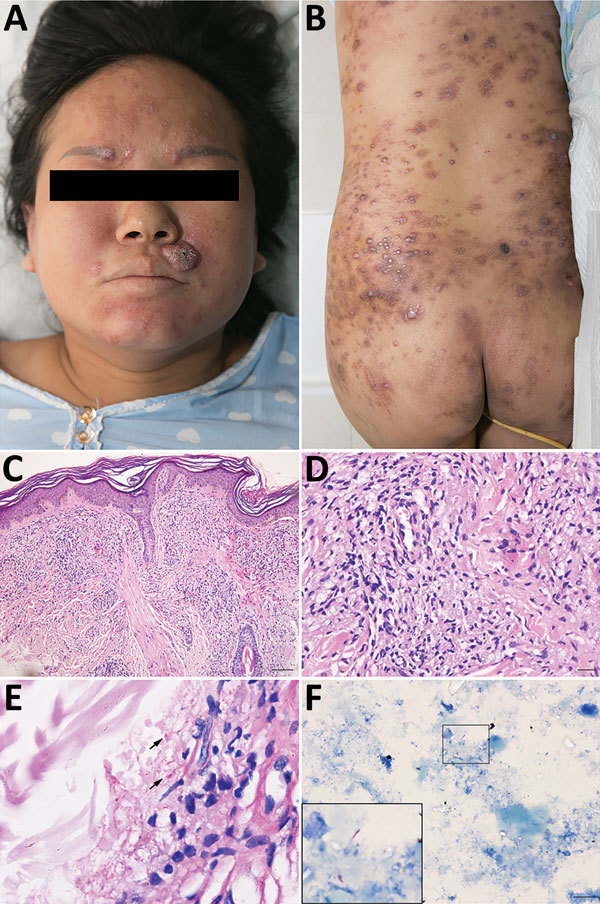
Clinical features of *Mycobacterium leprae* infection in pregnant woman and pathologic characteristics of a biopsy and placenta samples, China, December 2017. A, B) multiple brown papules and firm nodules on the woman’s trunk and face and ichththyosis presentation on the anterior tibia. C, D) Testing of biopsy sample from the face demonstrates subepidermal clear zone, nodular proliferation of spindle-shaped histiocytes in the dermis. Hematoxylin and eosin stain; original magnification ×10 (C) and ×40 (D). E) Numerous acid-fast bacilli in dermis (arrows). Acid-fast stain; original magnification ×100. F) Intact rod-shaped *M. leprae* from placenta homogenate; inset shows larger view. Acid-fast stain, original magnification ×100.

The patient refused treatment, citing concern about adverse effects on the fetus. Her condition was monitored with ultrasounds at serial intervals. At 37 weeks’ gestation, her amniotic membranes ruptured. She was transferred to an isolated operating room and underwent a cesarean delivery. She delivered a healthy baby girl. At the patient’s request, she was housed separately from her infant, and she decided not to breast-feed. After delivery, the patient was treated with dapsone, rifampin, and clofazimine, in accordance with World Health Organization recommendations (*2*).

After delivery, we collected fresh samples from the patient, including breast milk, umbilical cord, umbilical cord blood, and placenta, as well as nasal mucosa swab and serum specimens from the patient, her newborn, and her elder daughter for bacterial and serologic analysis. Intact acid-fast bacilli were found in placenta homogenates from the patient (Figure, panel F; Appendix Figure). Serologic testing for NDO, MMP-II, and LID antibodies by ELISA were all positive in the patient, whereas only MMP-II and LID antibodies were found in the newborn (Appendix Table 1). We also conducted PCR testing of various samples; some results were positive for the mother and her elder daughter, but none were positive for the newborn (Appendix Table 2). One month later, serologic test results for the infant were almost negative for *M. leprae* antibodies (Appendix Table 3). The patient’s lesions resolved, and her family members were shown to be healthy during follow-ups.

Leprosy can be exacerbated during pregnancy and, without treatment, can cause permanent damage to the skin, nerves, and eyes because of suppression of cell-mediated immunity in pregnancy. Downgrading reactions can occur, especially in the third trimester (*3*). Therefore, treating leprosy during pregnancy is critical. For multibacillary leprosy patients, World Health Organization treatment guidelines recommend multidrug therapy using rifampin, dapsone, and clofazimine (*2*). These agents must never be used alone as monotherapy for leprosy nor be stopped during pregnancy (*4*). 

Our patient refused treatment, citing concerns for adverse effects on the fetus; consequently, her condition dramatically worsened during the third trimester. Fortunately, no nerves or important organs were damaged. The patient’s breast milk was negative for DNA, RNA, and antibodies of *M. leprae*. Serum samples from umbilical cord blood were positive for DNA and IgG of *M. leprae* but negative for RNA and IgM. Notably, a substantial number of *M. leprae* organisms were detected in the placenta (Figure, panel F; Appendix Figure). 

Our findings support the assumption that the placental barrier can effectively stop vertical transmission of leprosy as well as the consensus that breast-feeding by women receiving multidrug therapy is safe for infants, given that no DNA or RNA of *M. leprae* were detected in breast milk (*5*,*6*). Although antileprosy drugs can be excreted into breast milk, no adverse effects have been reported except skin discoloration in the infant because of clofazimine (*7*). The patient’s elder daughter’s serum sample and nasal mucosa swab specimen were positive for *M. leprae* DNA and RNA by PCR, confirming that she was an *M. leprae* carrier. Households experiencing such a situation need to be screened with regular follow-ups (*8*).

Appendix Additional information regarding intact *Mycobacterium leprae* isolated from placenta of a pregnant woman, China.
