# Supplementary material for: Intact Mycobacterium leprae Isolated from Placenta of a Pregnant Woman, China
Source: Emerg Infect Dis. 2019 Aug;25(8):1604–7. doi: 10.3201/eid2508.190114 (PMC6649342; doi:10.3201/eid2508.190114)
Supplement: Appendix — Additional information regarding intact Mycobacterium leprae isolated from placenta of a pregnant woman, China. [file 19-0114-Techapp-s1.pdf]

Article DOI: https://doi.org/10.3201/eid2508.190114

Intact *Mycobacterium leprae* Isolated from Placenta of a Pregnant Woman, China

Appendix

Methods

Sample Collection and Preparation

Samples were obtained from the patient and his family members with informed consent. Sample for pathologic examination was fixed in 10% neutral buffered formalin and then sectioned in paraffin blocks for HE and AFB special stains. Placenta sample for mycobacteria AFB special stains was homogenized with glass pestle in 0.9% NS. Nasal secretion samples for PCR detection were collected as previously described (*1*). A sterile swab was carefully introduced into the antero-superior portion of one of the nostrils with a delicate swivel movement and lateral slip through the nasal wing. The procedure was repeated in the other nostril with the same swab, after which it was inserted in a microtube with the preservative TE 1X. The stem was cut with a scissors, enough to close the microtube. Saliva samples were collected by using Salivette Tube System (Sarstedt, Germany) according to the manufacturer's instructions.

Determining Antibody Responses by ELISA

NDO-BSA and LID-1 were generated at Infectious Disease Research Institute, Seattle, USA and MMP-II was generated at Department of Mycobacteriology, Leprosy Research Centre, National Institute of Infectious Diseases, Japan. ELISA for the detection of antigen-specific antibodies was performed in accordance with published procedures (*2*–*5*). The cutoff values were determined by Receiver Operating Characteristic (ROC) curve analysis of three replicate experiments as the value providing best overall performance characteristics for each antigen (sensitivity, specificity and area under the curve) (*6*). The cutoff values were defined as OD 450nm of 0.236, 0.165 and 0.138 for NDO-BSA, MMP-II and LID-1, respectively.

Reverse Transcription PCR Amplification of 16S rRNA and Gene Amplification of 16S rRNA, RLEP, and *folP1*

Before isolation of genomic DNA and RNA, oral mucosa, nasal mucosa, serum and breast milk samples were centrifuged at high speed, 13000 g for 20 min while the biopsy and placenta homogenate samples were directly used. Total RNA and DNA were simultaneously isolated using a same kit, All DNA/RNA Mini Kit according to manufacturer's instructions (CAT #:80204, Qiagen, Germany). The Obtained RNA was subjected to reverse transcription -PCR of 16S rRNA and further subjected to PCR amplification by using specific primers and conditions described elsewhere (*7*). The presence 16S rRNA, RLEP and *folP*1 genes of *M. leprae* were detected by using the primers and conditions described previously (*8*,*9*). All the amplified products were analyzed with 1.5% agarose gels.

References

1. Carvalho RS, Foschiani IM, Costa MRSN, Marta SN, da Cunha Lopes Virmond M. Early detection of *M. leprae* by qPCR in untreated patients and their contacts: results for nasal swab and palate mucosa scraping. Eur J Clin Microbiol Infect Dis. 2018;37:1863–7. PubMed http://dx.doi.org/10.1007/s10096-018-3320-9

2. Wang H, Liu W, Jin Y, Yu M, Jiang H, Tamura T, et al. Detection of antibodies to both *M. leprae* PGL-I and MMP-II to recognize leprosy patients at an early stage of disease progression. Diagn Microbiol Infect Dis. 2015;83:274–7. PubMed http://dx.doi.org/10.1016/j.diagmicrobio.2015.07.012

3. Wu Q, Yin Y, Zhang L, Chen X, Yu Y, Li Z, et al. A study on a possibility of predicting early relapse in leprosy using a ND-O-BSA based ELISA. Int J Lepr Other Mycobact Dis. 2002;70:1–8. PubMed

4. Duthie MS, Goto W, Ireton GC, Reece ST, Cardoso LPV, Martelli CMT, et al. Use of protein antigens for early serological diagnosis of leprosy. Clin Vaccine Immunol. 2007;14:1400–8. PubMed http://dx.doi.org/10.1128/CVI.00299-07

5. Duthie MS, Balagon MF, Maghanoy A, Orcullo FM, Cang M, Dias RF, et al. Rapid quantitative serological test for detection of infection with *Mycobacterium leprae*, the causative agent of leprosy. J Clin Microbiol. 2014;52:613–9. PubMed http://dx.doi.org/10.1128/JCM.02085-13

6. Le W, Haiqin J, Danfeng H, Ying S, Wenyue Z, Jun Y, et al. Monitoring and detection of leprosy patients in southwest China: a retrospective study, 2010-2014. Sci Rep. 2018;8:11407. PubMed http://dx.doi.org/10.1038/s41598-018-29753-4

7. Jadhav RS, Kamble RR, Shinde VS, Edward S, Edward VK. Use of reverse transcription polymerase chain reaction for the detection of *Mycobacterium leprae* in the slit-skin smears of leprosy patients. Indian J Lepr. 2005;77:116–27. PubMed

8. Yoon KH, Cho SN, Lee MK, Abalos RM, Cellona RV, Fajardo TT Jr, et al. Evaluation of polymerase chain reaction amplification of *Mycobacterium leprae*-specific repetitive sequence in biopsy specimens from leprosy patients. J Clin Microbiol. 1993;31:895–9. PubMed

9. World Health Organization. Guidelines for global surveillance of drug resistance in leprosy [cited 2019 Feb 20]. https://apps.who.int/iris/handle/10665/205158

Appendix Table 1. ELISA based detection of antibody at the time of delivery

Sample	NDO-BSA (IgM)	MMP-II (IgG)	LID-1 (IgG)
Blank	0.056	0.067	0.066
Positive control	0.790	0.634	0.381
Negative control	0.067	0.076	0.065
Patient	0.467	0.571	0.220
Umbilical Cord	0.054	0.515	0.142

Appendix Table 2. Results of PCR detection of patient and household samples*

	Patient					Elder daughter			Newborn		
Sample	OM	NM	Se	BM	P	OM	NM	Se	OM	NM	Se
16S rRNA(RNA)†	+	+	+	–	+	+	–	–	–	–	–
16S rRNA (DNA)‡	+	+	+	–	+	–	–	–	–	–	–
RLEP	+	+	+	–	+	+	+	–	–	–	–

*BM, breast milk; NM, nasal mucosa; OM, oral mucosa; P, placenta; Se, serum; +, positive; –, negative.
†cDNA as template.
‡Genomic DNA as template.

Appendix Table 3. ELISA based detection of antibody at 1-month post-delivery

Sample	NDO-BSA (IgM)	MMP-II (IgG)	LID-1 (IgG)
Blank	0.048	0.062	0.067
Positive control	0.742	0.646	0.371
Negative control	0.053	0.061	0.062
Patient	0.631	0.557	0.201
Newborn	0.057	0.187	0.188

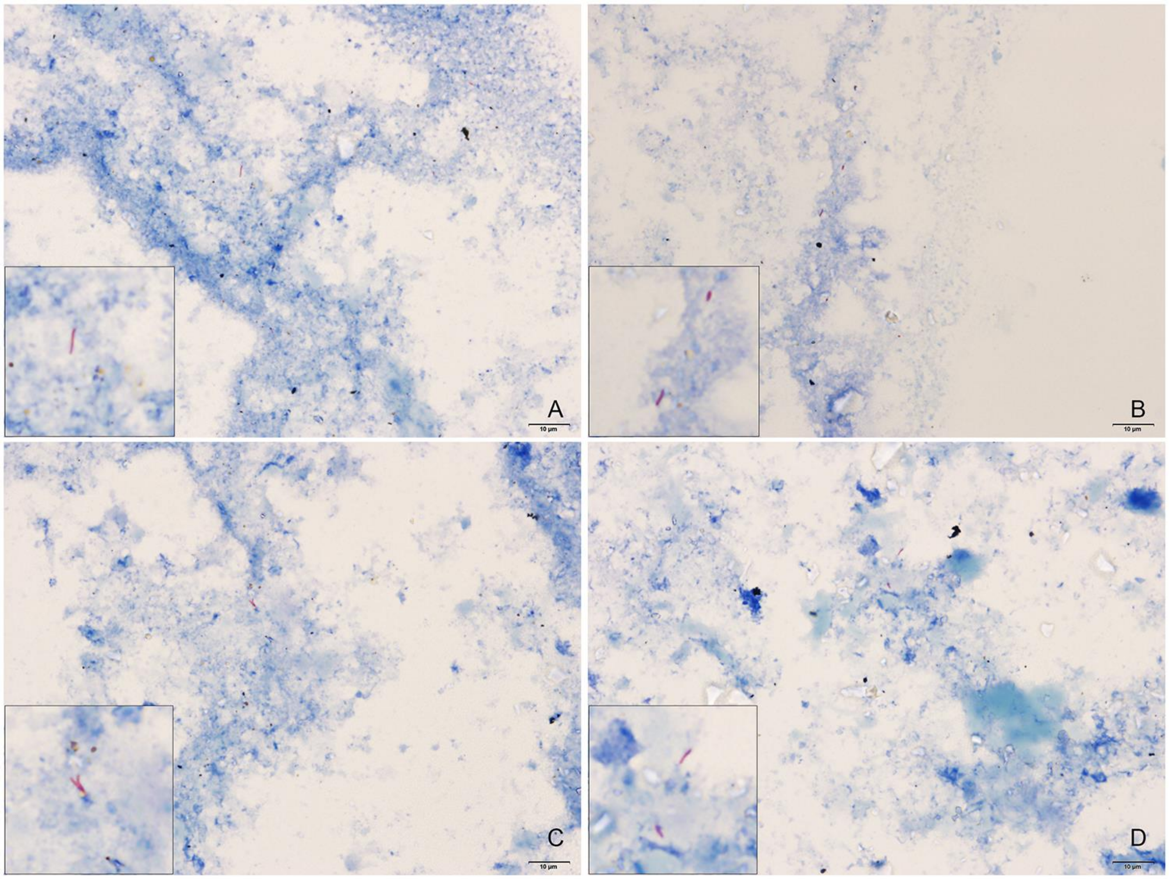

Appendix Figure. Microscopic examination of intact rod-shaped *Mycobacterium leprae* bacilli from placenta homogenate as square box zoom in (acid-fast stain, original magnification × 400).
